# Impact of transforaminal epidural steroid injection on pain, disability, and kinesiophobia in lumbar disc herniation with radicular pain

**DOI:** 10.55730/1300-0144.6203

**Published:** 2026-02-12

**Authors:** Hande Ece ÖZ, Rumeysa ÇETİNKAYA BULUTOĞLU, Furkan ÇETİN, Ezgi CAN, Alp Eren ÇELENLİOĞLU, Ender SİR

**Affiliations:** Department of Algology, Faculty of Medicine, Gülhane Training and Research Hospital, University of Health Sciences, Ankara, Turkiye

**Keywords:** Lumbar epidural injections, kinesiophobia, radicular pain

## Abstract

**Background/aim:**

The aim was to evaluate the effect of single-level transforaminal epidural steroid injection (TFESI) on kinesiophobia (KP) in patients with lumbar radicular pain (RP) caused by single-root, single-level lumbar disc herniation (LDH).

**Materials and methods:**

A total of 63 patients diagnosed with unilateral single-level radicular pain due to LDH and treated with single-level TFESI were included. The Numeric Rating Scale (NRS), modified Oswestry Disability Index (MODI), Tampa Scale of Kinesiophobia (TSK), and Neuropathic pain (NP)-Douleur Neuropathique 4 Questionnaire (DN4) were used before the procedure and at 3 weeks and 3 months postoperatively.

**Results:**

At baseline, 76.19% (n = 48) of the patients exhibited KP. Significant improvements were observed in the NRS, TSK, MODI, and DN4 scores at both 3 weeks and 3 months (p < 0.001). Clinical outcomes were similar between the patients with and without KP, except for DN4 scores, which remained significantly higher in the KP group. A ≥50% reduction in the TSK score at the 3-month follow-up was positively correlated with ≥50% improvements in the NRS, MODI, and DN4 scores. Additionally, a longer symptom duration was negatively associated with the degree of improvement in the TSK.

**Conclusion:**

Our findings suggest that TFESI is an effective and safe method for alleviating pain and kinesiophobia in patients with chronic radicular pain due to LDH. Clinicians should be aware that a prolonged duration of symptoms is a risk factor that negatively influences the effectiveness of TFESI in cases of KP.

## 1. Introduction

Lumbar disc herniation (LDH) is a common cause of low back and leg pain [1]. Compression or irritation of the lumbar spinal nerve roots by a herniated lumbar disc causes radicular pain (RP), lower limb numbness, muscle weakness, and even incontinence in severe cases [2]. Low back pain with RP due to LDH is associated with higher levels of pain, increased disability, higher frequency of healthcare utilization, and medical interventions than isolated lower back pain [3].

Conservative treatments for LDH include nonsteroidal antiinflammatory drugs, physical therapy, lifestyle modifications, and corticosteroid injections for selected cases [4]. Transforaminal epidural steroid injection (TFESI) is a widely used intervention for managing RP due to LDH in patients who remain symptomatic despite conservative treatment [5,6]. In clinical practice, TFESI-based interventional approaches have been associated with significant pain relief and improved function in patients with RP due to LDH [7,8]. Epidural steroid injections have also been reported to improve pain and functional outcomes in other degenerative lumbar conditions, supporting their broader role as an interventional option when conservative measures fail [9].

Kinesiophobia (KP) is defined as excessive and irrational fear of movement, often driven by concerns about pain or the chance of reinjury [10]. The prevalence of KP can be as high as 80% in specific groups with conditions such as chronic low back pain [11]. These findings suggest that patients with LDH are prone to developing KP, which can complicate their recovery [12]. Fear-driven avoidance of movement can lead to reduced physical activity, muscle deconditioning, and a self-reinforcing cycle with KP in clinical practice, and so it is essential to prevent it for improving recovery outcomes and enhancing the overall health of patients with LDH [11].

Our primary objective was to investigate the effect of TFESI on KP in patients with unilateral single-level LDH and RP. Our secondary aim was to evaluate the impact of KP before the procedure on the TFESI treatment outcomes.

## 2. Materials and methods

### 2.1. Patients and methods

This prospective clinical study was conducted at our clinic between March 2025 and July 2025. All patients were informed about the study procedure and written informed consent was obtained. The local ethics committee approved this prospective study (approval number: 2024-KAEK-40). The study was conducted in accordance with the principles of the Declaration of Helsinki.

Prior to being referred to our clinic, every patient had undergone a minimum of 4 weeks of medical and conservative treatment in accordance with the guidelines. Conservative treatment includes rest during acute exacerbations, activity modification, structured physical therapy, and exercise programs. Nonsteroidal antiinflammatory drugs, analgesics, and muscle relaxants were used for medical treatment. Patients with moderate-to-severe radicular pain of moderate to severe levels despite undergoing these treatments were assessed for eligibility. Those who met the inclusion criteria were enrolled.

The inclusion criteria were as follows: age between 18 and 75 years, LBP with unilateral pain radiating to the lower extremities for 1 month or more and not responding to conservative treatment modalities, not taking gabapentin or pregabalin for neuropathic pain, Numeric Rating Scale (NRS) score of ≥4, unilateral lumbar RP on physical examination, and LDH at L3–4, L45, or L5S1 levels with compression of the L4, L5, or S1 spinal nerve root within the previous 6 months, as shown by magnetic resonance imaging (MRI). Patients with bilateral or multisegmental symptoms, prior lumbar surgery (lumbar fusion or laminectomy), transitional vertebrae, lumbar spinal stenosis, spondylolysis, spondylolisthesis, scoliosis, lumbar epidural steroid injection within the previous 3 months, bleeding diathesis, or systemic or local infections were excluded from the study.

**Figure 1:**
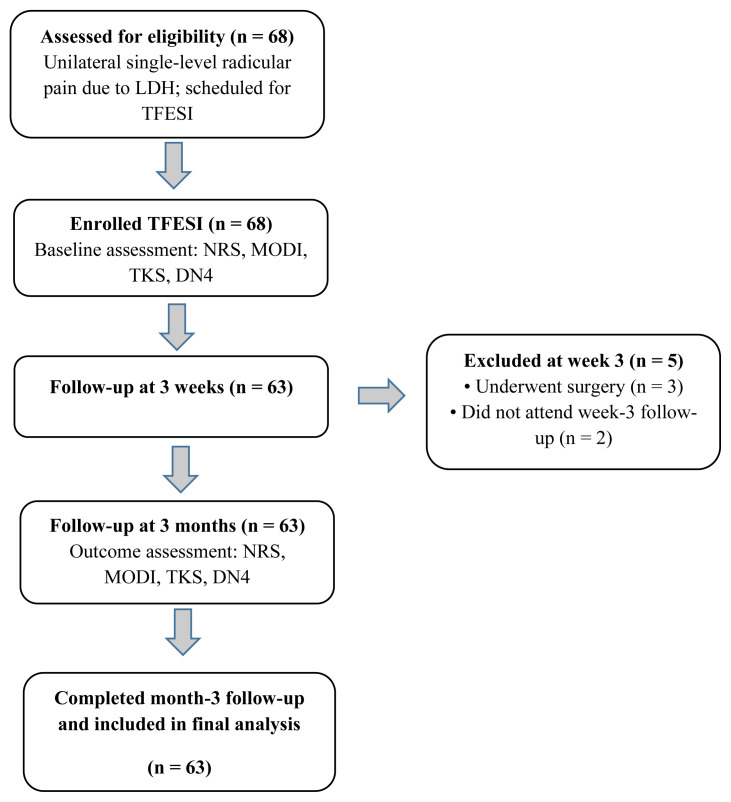
Flow diagram of patient selection and follow-up.

The study included 68 patients with LBP and unilateral pain radiating to the lower extremities (Figure 1). Patients diagnosed with single-level RP due to LDH based on clinical signs, physical examination, and MRI findings and unresponsive to conservative treatment were scheduled for single-level TFESI.

Fluoroscopy-guided TFESI was performed by the same pain medicine specialist with at least 10 years of experience. Assessments were conducted prior to the interventional treatment and 3 weeks and 3 months following the TFESI. Age, sex, height, weight, body mass index (BMI), comorbid medical conditions (e.g., diabetes mellitus and hypertension), duration of pain, and target level of the affected nerve root were recorded before the interventional treatment. The 10-point NRS (NRS-10), where 0 indicates no pain and 10 represents unbearable pain, was used to assess RP severity.

Physical function and disability were evaluated using the Turkish version of the modified 10-item Oswestry Disability Index (MODI), which ranges from 0 to 100, with higher scores indicating greater disability [13]. Neuropathic pain (NP) was assessed using the Douleur Neuropathique 4 (DN4) questionnaire. The DN4 consists of seven items related to pain characteristics (burning, painful cold, electric shocks, tingling, pins and needles, numbness, and itching) and three items based on physical examination (hypoesthesia to brushing, hypoesthesia to light touch, and brush-induced allodynia) [14]. We used the Turkish version of the TSK, which is a 17-item self-report questionnaire that assesses the subjective level of KP, with scores ranging from 17 to 68 to evaluate KP [15]. High kinesiophobia is operationally defined as TSK scores exceeding 37, in alignment with Vlaeyen’s foundational framework and subsequent empirical validations [10]. All questionnaires were administered to the patients before the procedure and at 3 weeks and 3 months postinjection.

### 2.2. TFESI technique

**Figure 2:**
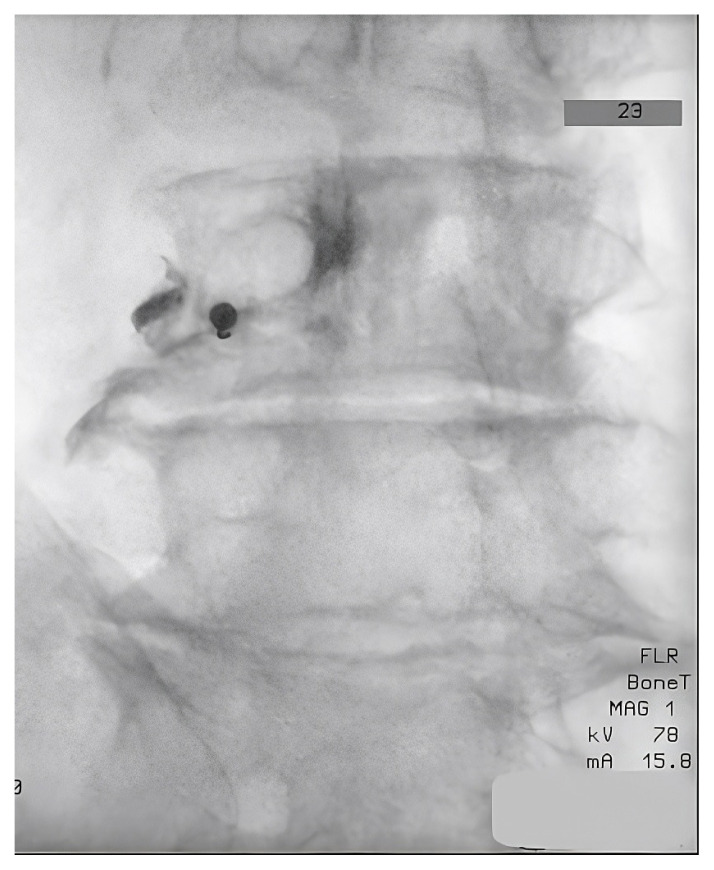
Fluoroscopic image showing contrast spread during the transforaminal epidural steroid injection.

The injection site was disinfected three times using povidone–iodine solution and then covered with a sterile cloth. The C-arm fluoroscopy machine was tilted at an angle ranging from 10 to 30 degrees to clearly visualize the intervertebral foramen. Local anesthetic was administered via 3 mL of 2% prilocaine to both the epidermis and subcutaneous layers. A 3.5-inch, 22-gauge Quincke spinal needle was then inserted into the subpedicular space, aligned in the 6 o’clock direction with intermittent fluoroscopic guidance using a coaxial method. The needle position was validated in the lateral view. Then, 1–2 mL of contrast dye was injected to confirm the epidural positioning, which was checked in both anteroposterior and lateral fluoroscopic views (Figure 2). After ensuring appropriate contrast dispersion and confirming the absence of vascular uptake, 8 mg of dexamethasone, 1 mL of 0.5% bupivacaine, and 1 mL of physiological saline mixture were administered. All participants were then observed for 1 h in the recovery room following the injection. Patients without postprocedural complications were discharged with relevant directives.

### 2.3. Statistical analysis

Owing to the lack of previous studies with a similar design, sample size calculation could not be performed at the beginning of the present study. Therefore, all patients who met the inclusion criteria during the study period were consecutively enrolled. All analyses were conducted using IBM SPSS Statistics for Windows, Version 23.0. The findings of our study are expressed as frequency and percentage. Normality analysis was performed using the Shapiro–Wilk test, skewness and kurtosis, and histograms. Categorical variables are presented as absolute numbers and percentages. Continuous variables were compared between the groups using the Mann–Whitney U-test and are presented as medians with interquartile ranges. Categorical variables were compared using the chi-squared or Fisher’s exact tests. NRS, MODI, TSK, and DN4 scores were compared using the Friedman test. The correlation coefficients (rho) between the variables were determined. Statistical significance was set at p < 0.05.

## 3. Results

In the present study, 68 patients were initially evaluated for eligibility; however, at the third week of follow-up, five patients were excluded because three of them had undergone surgery and two did not attend the third week follow-up. The final analysis involved a total of 63 patients who successfully completed the follow-up assessment. No major complications were observed, except for a mild vasovagal reaction during the procedure in three patients. No significant complications were observed during the postoperative period following the procedure.

The total number of patients was 63, of whom 27 were men and 36 were women. The median duration of pain experienced by the patients was 6 months. The most frequently targeted nerve root level for TFESI was L5 (53.9%), followed by L4 (44.4%) and S1 (1.7%) levels. At baseline, 48 patients (76.19%) were diagnosed with KP (Table 1).

Statistical analysis demonstrated substantial improvements in all measured outcomes (NRS, TSK, MODI, and DN4) at all postintervention assessment points (Table 2). The mean TSK scores decreased from 43.1 ± 9.7 at the initial baseline to 23.1 ± 2.0 and 25.8 ± 11.1 in the respective follow-up periods (Table 2). A reduction in NRS of at least 50% was commonly used as a clinically meaningful benchmark. This responder analysis was considered exploratory and supportive of continuous outcome findings.

The patients were categorized into two distinct groups based on the presence of KP at baseline (TSK ≥ 37 versus TSK < 37; Table 3). The initial comparison of sociodemographic and clinical factors revealed no significant differences between the two groups (Table 4). Both groups demonstrated significant progress in the NRS, MODI, and DN4 scores at all time points after treatment (p < 0.001, Table 3). In addition, the ratios of patients achieving a ≥50% enhancement in NRS, MODI, and DN4 metrics at 3 months were comparable between the two groups (p > 0.05, Table 3). However, DN4 levels were consistently elevated in the KP cohort at baseline, 3 weeks, and 3 months (p = 0.003, 0.032, and 0.005, respectively; Table 3).

The results of the correlation analysis demonstrated a statistically significant inverse relationship between the duration of symptoms and a ≥50% decrease in TSK scores at 3 months (Table 5). Furthermore, a minimum enhancement of 50% in TSK scores was positively correlated with a minimum reduction of 50% in the NRS (r = 0.508, p < 0.001), MODI (r = 0.400, p = 0.001), and DN4 (r = 0.363, p = 0.003) scores at 3 months. No statistically significant correlations were identified between the observed improvements in TSK scores and age (r = 0.003, p = 0.978) or BMI (r = 0.110, p = 0.390).

## 4. Discussion

The primary aim of the present study was to assess the prevalence of KP in patients with single-level RP due to LDH. While KP’s impact on spine pain is recognized, data on its changes post-TFESI in LDH are scarce. Our results suggest that fear of movement may improve following TFESI, consistent with fear-avoidance models and recent psychometric analyses of the TSK [16,17]. At baseline, a significant proportion of the patients (n = 48, 76.19%) had a high TSK. Postprocedure TSK scores significantly decreased at both the 3-week and 3-month assessments, parallel with the improvements in the NRS, MODI, and DN4 scores. Additionally, treatment responses were comparable between the patients with and without KP at all time points.

TFESI can provide significant short-term pain relief and functional improvement in patients with lumbar RP due to LDH [7]. Although long-term outcomes are not always consistent, some studies suggest that TFESI can provide substantial pain relief and improved functional outcomes over 6 months [7]. Our findings were consistent with the current literature, supporting that TFESI is associated with short-term improvements in pain and function in lumbar RP due to LDH.

KP is prevalent among patients with chronic low back pain, with prevalence rates ranging from 70% to 82% in various contexts [18–20]. The prevalence of KP in such patients is influenced by several factors, including symptom duration, pain intensity, and functional disability [18,19]. The influence of age, sex, and BMI on KP has been demonstrated [20]. Our study revealed that a significant proportion of the participants (n = 48, 76.19%) with lumbar RP due to LDH also had high levels of TSK.

KP, characterized by increased pain perception, reduced physical function, and decreased range of motion, has been shown to result in prolonged recovery times and suboptimal rehabilitation outcomes. Furthermore, it has been shown to have a significant impact on surgical outcomes in patients with lumbar RP [12,21]. A study in patients who underwent surgery for LDH revealed that a significant proportion of the participants exhibited symptoms of KP. This psychological concern was found to be associated with a deterioration in various domains of functioning, including disability, pain, health-related quality of life, depression, pain catastrophizing, and self-efficacy [12]. Nevertheless, the findings of the present study show that, following TFESI, patients reported diminished apprehension of movement, in conjunction with NRS and MODI scores. Our study revealed a negative correlation between symptom duration and TSK. TFESI may be associated with substantial symptomatic improvement. Therefore, early effective interventional treatment may help limit the development of KP.

Individuals with CLBP and KP have the potential for exacerbated pain perception, increased disability, and delayed recovery. Multifaceted psychological and complex physiological mechanisms can be responsible for the development of KP in this patient group, with the potential to increase disability and pain intensity over time [22,23]. The fear-avoidance belief system, pain catastrophizing, and low self-efficacy are further psychological factors that have been demonstrated to contribute to this condition [24]. In addition to psychological factors, physiological factors should also be considered, including elevated pain levels, distorted perception of pain, and altered sensory qualities of pain [11]. Impaired proprioception and reduced muscle endurance, particularly in the lumbar extensors, have been demonstrated to reinforce the fear of movement by increasing the perceived risk of injury during physical activity [25,26].

In the present study, baseline DN4 scores were significantly higher in the KP cohort. Furthermore, an improvement in the TSK scores of more than 50% at 3 months exhibited a statistically significant positive correlation with improvements greater than 50% in the DN4 scores. This finding may indicate a possible correlation between neuropathic pain symptoms and maladaptive fear-based behavior.

The notable negative correlation observed between symptom duration and KP improvement suggests that chronicity can exacerbate maladaptive beliefs and behaviors. This observation is consistent with the findings of previous investigations, which suggest that persistent pain may result in central sensitization and psychological reinforcement of fear-avoidance behaviors [25]. Therefore, early strategies could be pivotal in addressing pain and preventing the development of chronic disabilities related to fear. It is crucial to understand and address the KP to enhance rehabilitation practices and improve patient outcomes.

The L4–L5 and L5–S1 levels are mostly affected by LDH; however, our study found a striking prevalence of L3–L4 herniation [27]. This observation likely stems from the referral patterns in our tertiary care facility, which directs patients with L5–S1 issues to surgical consultation earlier than other facilities. The higher proportion of L3–L4 involvement in our cohort may be due to the referral patterns and case load in our tertiary care center, which frequently evaluates patients with more complex or atypical presentations of radiculopathy. Furthermore, the significant baseline pain intensity and lengthy median symptom duration in our cohort accentuate the frequent practice of extended conservative management prior to patient referral. Consistent with previous reports demonstrating the effectiveness of epidural injections in chronic and severe radicular pain, our results indicate that TFESI can significantly enhance pain relief, disability, and movement apprehension, even in patients with enduring severe symptoms [28].

The limitations of the present study can be attributed to several factors. First, the absence of a comparator group limited causal interpretation of the observed changes. Furthermore, the study was conducted at a single tertiary care center, which limited the generalizability of the results. The relatively modest sample size and the relatively modest follow-up duration were further limitations. Additionally, the absence of a priori sample size calculations and the relatively small subgroup sizes may have reduced the statistical power to detect differences between groups, particularly in comparisons between patients with and without baseline kinesiophobia. The quantification of supervised physiotherapy or physical activity during the symptomatic phase was not systematically conducted, allowing for potential residual confounding factors from prior rehabilitation. This limitation was partially addressed by adjusting for baseline disability, neuropathic pain characteristics, and symptom duration. Finally, we did not assess other psychosocial constructs, which may also have influenced our findings.

In conclusion, our findings indicate that TFESI was associated with improvements in pain and KP components in individuals with chronic RP due to LDH. It is crucial for practitioners to recognize that KP frequently acts as a significant and recurrent factor that negatively impacts patients and requires consideration throughout the treatment process. Moreover, these findings suggest that KP may not exclusively constitute a psychological comorbidity but a component of the pain experience that may improve following interventional treatment.

## Figures and Tables

**Table 1:** Demographic and clinical features of the patients and basal scale scores.

Variables (n=63)	Results
Age (median, min–max / mean±SD)	52 (25–73) / 52.4±11.6
Pain duration, months (median, min–max / mean±SD)	6 (1–36) / 8.4±6.7
Body mass index (median, min–max / mean±SD)	28.4 (18.8–53.2) / 29.6±5.8
Sex, n (%)	
Female	36 (57.1 %)
Male	27 (42.9 %)
Treated nerve root level, n (%)	
Lumbar–4	28 (44.4 %)
Lumbar–5	34 (53.9 %)
Sacral–1	1 (1.7 %)
Basal NRS scores (median, min–max / mean±SD)	8 (5–10) / 8.2±1.3
Basal TSK scores (median, min–max / mean±SD)	42 (17–63) / 43.1±9.7
Basal MODI scores (median, min–max / mean±SD)	42 (11–94) / 46.9±23.1
Basal DN4 scores (median, min–max / mean±SD)	4 (0–9) / 4.1±2.2

Data are expressed as median values (min–max) or number of patients (%), SD: Standard deviation, NRS: Numerical rating scale, MODI: Modified Oswestry Disability Index, TSK: Tampa Scale for Kinesiophobia, DN4: 4-question neuropathic pain questionnaire.

**Table 2:** Pre- and postprocedural NRS, TSK, MODI, and DN4 scores of the patients.

		Mean±SD	Median (min–max)	Mean rank	p-value
NRS	Basal	8.2**±**1.3	8 (5–10)	2.98	**<0.001** ^*^
	Week 3	3.2**±**2.1	3 (0–8)	1.47	
	Month 3	3.3±2.3	3 (0–9)	1.56	
TSK	Basal	43.1**±**9.7	42 (17–63)	2.96	**<0.001** ^**^
	Week 3	23.1±2.0	20 (12–50)	1.47	
	Month 3	25.8±11.1	21 (8–52)	1.57	
MODI	Basal	46.9**±**23.1	42 (11–94)	2.92	**<0.001** ^**^
	Week 3	23.1±16.1	18 (0–78)	1.52	
	Month 3	22.6±13.8	20 (0–56)	1.56	
DN4	Basal	4.1**±**2.2	4 (0–9)	2.67	**<0.001** ^**^
	Week 3	2.3±1.5	2 (0–7)	1.62	
	Month 3	2.4±1.6	2 (0–7)	1.71	

Friedman Test. Data are expressed as median values (min–max) or number of patients (%), SD: Standard deviation, NRS: Numerical rating scale, MODI: Modified Oswestry Disability Index, TSK: Tampa Scale for Kinesiophobia, DN4: 4-question neuropathic pain questionnaire. Bold p-values indicate statistical significance.

**Table 3:** Pre- and postprocedural NRS, MODI and DN4 scores according to the presence of kinesiophobia.

	Kinesiophobia		
	Yes (TSK≥37) No (TSK<37)		
Variables	(n=48) (n=15)		
	Mean±SD/mean rank	Mean±SD/mean rank	p-value
NRS-Basal	8 (5–10) / 30.5	8 (7–10) / 36.5	0.257*
NRS-3rd week	3 (0–8) / 31.9	3 (0–5) / 32.1	0.993*
NRS-3rd month	3 (0–9) / 32.9	2 (0–6) / 29.1	0.472*
p-value	**<0.001** **	**<0.001** **	
MODI-Basal	45 (11–94) / 33.6	36 (14–68) / 26.7	0.199*
MODI-3rd week	18 (0–78) / 33.6	18 (2–46) / 27.0	0.225*
MODI-3rd month	22 (0–56) / 34.3	14 (3–42) / 24.8	0.079*
p-value	**<0.001** **	**<0.001** **	
DN4-Basal	4.5 (0–9) / 35.7	3 (0–7) / 20.0	**0.003** *
DN4-3rd week	2 (0–7) / 34.7	2 (0–5) / 23.4	**0.032** *
DN4-3rd month	2.5 (0–7) / 34.5	2 (0–5) / 24.1	**0.005** *
p-value	**0.021** **	**<0.001** **	

* Mann–Whitney U Test,

** Friedman Test, NRS: Numerical rating scale, MODI: Modified Oswestry Disability Index, TK: Tampa Scale for Kinesiophobia, DN4: 4-question neuropathic pain questionnaire. Bold p-values indicate statistical significance.

**Table 4:** Sociodemographic and clinical characteristics according to the presence of kinesiophobia.

	Kinesiophobia		
	Yes (TSK≥37) No (TSK<37)		
Variables	(n=48) (n=15)		
	Median (min–max)	Median (min–max)	p-value
Age	51 (25–71)	55 (30–73)	0.457*
Body mass index	29.2 (18.8–53.2)	26.7 (22–37)	0.110*
Pain duration (months)	7 (1–36)	5 (1–24)	0.231
Basal NRS scores	8 (5–10)	8 (7–10)	0.257*
Basal MODI scores	45 (11–94)	36 (14–68)	0.199*
Basal DN4 scores	4.5 (0–9)	3 (0–7)	**0.003** *
Sex	n (%)	n (%)	
Female	28 (58.3)	8 (53.3)	0.966^a^
Male	20 (41.7)	7 (46.7)	
Treated nerve root level			
L 4	21 (43.7)	7 (46.6)	0.121^b^
L 5	27 (56.3)	7 (46.6)	
S 1	0 (0)	1 (6.8)	
≥50% relief in NRS-3m			
Yes	34 (70.8)	12 (80.0)	0.740^c^
No	14 (29.2)	3 (20.0)	
≥50% relief in MODI-3m			
Yes	23 (47.9)	10 (66.7)	0.331^a^
No	25 (52.1)	5 (33.3)	
≥50% relief in DN4-3m			
Yes	21 (43.7)	5 (33.3)	0.678^a^
No	27 (56.3)	10 (66.7)	

* Mann–Whitney U test,

a Continuity correction test,

b Pearson chi-squared test,

c Fisher’s exact test,

NRS: Numerical rating scale, MODI: Modified Oswestry Disability Index, TSK: Tampa Scale for Kinesiophobia, DN4: 4-question neuropathic pain questionnaire. Bold p-values indicate statistical significance.

**Table 5:** Correlations between **≥**50% relief in TSK scores and other factors.

	≥50% relief in TSK-3rd month scores	
Variables	r	p
Age, years	0.003	0.978
Body mass index	0.110	0.390
Pain duration, months	−0.329	**0.008**
≥50% relief in NRS-3rd month	0.508	**<0.001**
≥50% relief in MODI-3rd month	0.400	**0.001**
≥50% relief in DN4-3rd month	0.363	**0.003**

Spearman correlation test, r: Rho, p: p-value, NRS: Numerical rating scale, MODI: Modified Oswestry Disability Index, TSK: Tampa Scale for Kinesiophobia, DN4: 4-question neuropathic pain questionnaire. Bold p-values indicate statistical significance.
